# Comparative Transcriptomic Analysis Reveals Similarities and Dissimilarities in *Saccharomyces cerevisiae* Wine Strains Response to Nitrogen Availability

**DOI:** 10.1371/journal.pone.0122709

**Published:** 2015-04-17

**Authors:** Catarina Barbosa, José García-Martínez, José E. Pérez-Ortín, Ana Mendes-Ferreira

**Affiliations:** 1 Centre of Agricultural Genomics and Biotechnology (CGBA) Universidade de Trás-os-Montes e Alto Douro, Vila Real, Portugal; 2 Universitat de Valência, Departamento de Genética y E.R.I. Biotecmed, València, Spain; 3 Universitat de Valência, Departamento de Bioquímica y Biología Molecular y E.R.I. Biotecmed, València, Spain; University of Strasbourg, FRANCE

## Abstract

Nitrogen levels in grape-juices are of major importance in winemaking ensuring adequate yeast growth and fermentation performance. Here we used a comparative transcriptome analysis to uncover wine yeasts responses to nitrogen availability during fermentation. Gene expression was assessed in three genetically and phenotypically divergent commercial wine strains (CEG, VL1 and QA23), under low (67 mg/L) and high nitrogen (670 mg/L) regimes, at three time points during fermentation (12h, 24h and 96h). Two-way ANOVA analysis of each fermentation condition led to the identification of genes whose expression was dependent on strain, fermentation stage and on the interaction of both factors. The high fermenter yeast strain QA23 was more clearly distinct from the other two strains, by differential expression of genes involved in flocculation, mitochondrial functions, energy generation and protein folding and stabilization. For all strains, higher transcriptional variability due to fermentation stage was seen in the high nitrogen fermentations. A positive correlation between maximum fermentation rate and the expression of genes involved in stress response was observed. The finding of common genes correlated with both fermentation activity and nitrogen up-take underlies the role of nitrogen on yeast fermentative fitness. The comparative analysis of genes differentially expressed between both fermentation conditions at 12h, where the main difference was the level of nitrogen available, showed the highest variability amongst strains revealing strain-specific responses. Nevertheless, we were able to identify a small set of genes whose expression profiles can quantitatively assess the common response of the yeast strains to varying nitrogen conditions. The use of three contrasting yeast strains in gene expression analysis prompts the identification of more reliable, accurate and reproducible biomarkers that will facilitate the diagnosis of deficiency of this nutrient in the grape-musts and the development of strategies to optimize yeast performance in industrial fermentations.

## Introduction

The commercial wine yeast strains selection is based on a range of phenotypic characteristics embracing fermentation and technological proprieties with high regard for the production of high quality wines [1]. The major requirements for a wine starter are its ability to grow and complete efficiently the conversion of grape sugars to ethanol, which are highly dependent on the nitrogen availability in grape-juice [2]. Yeast nitrogen requirements are reliant on the amount of sugars present, the higher the initial sugar concentration the more the nitrogen will be needed to complete sugar fermentations [3]. Additionally, improved ethanol tolerance of yeast has been associated with higher initial nitrogen concentrations by the stimulation and maintenance of maximum specific growth rate [2, 4] and by the increased storage of nitrogen compounds in the vacuoles, important for maintenance of yeast metabolic activity in the latter fermentation stages, where high ethanol levels unable efficient nitrogen transport [5]. Nevertheless, it is well known that wine strains display a huge diversity in their nitrogen demands and these differences are reflected in their fermentative activity [2, 6–8].

Yeast strain genetic variation in gene expression has been associated with phenotypic diversity [9–11]. The genome-wide transcriptional response of wine yeast during alcoholic fermentation has been a topic of detailed investigations in the recent years [12–19]. All these studies have shown the genome plasticity of wine yeasts revealing common transcriptional features. Overall, genes involved in C-compound metabolism, mitochondrial respiration, oxidative stress and stress responsive genes [20] are up-regulated, while genes primarily involved in cell growth, protein biosynthesis and ribosomal processing, functions are repressed during alcoholic fermentation progression. Marks *et al*. [18] even identified a set of genes, designated as Fermentation Stress Response (FSR) genes that are induced throughout fermentation and are considered to mediate long-term adaptation to the increasing ethanol levels. A few studies have focused on the comparative analysis of gene expression among wine strains under nutrient sufficient conditions [21–23]. These works have demonstrated that gene expression variability among strains is a source of phenotypic diversity.

The effect of initial nitrogen availability on gene expression profiles has also been evaluated, by comparing low and high nitrogen conditions and using distinct nitrogen sources [7, 12, 17]. These studies indicated that during nitrogen limited fermentations there is an increased expression of genes involved in oxidative metabolism and in ribosome adjustment, irrespective of the strain and nitrogen source used. The increased expression of the genes involved in respiration and oxidative stress response has been recently associated with improved fermentative activity of yeast cells under either in low nitrogen [7] or in nitrogen sufficient fermentations [24]. This information about how yeast cells respond to the stress conditions, particularly low assimilable nitrogen, has provided valuable data of practical interest for the control and prevention of slow and premature fermentation arrest during winemaking. Actually, there has been a great effort to identify genes whose expression responds to nitrogen limitation under enological conditions and that could be used as biomarkers for predicting nitrogen deficiency. Genes such as *CAR1* [25], *ACA1* [26], *FSP2*, *RGS2*, *AQY1*, *AGX1* [12], *DAL4* and *GAP1* [27], *ICY1* [28] has been pointed as good indicators of nitrogen nutritional state. Also a previous study conducted in our laboratory has identified thirty-six nitrogen responsive genes by comparing a nitrogen replete with several nitrogen depleted conditions, throughout fermentation [29]. However these sets of genes slightly overlap suggesting a lack of common signature between strains, conditions used, and timings of assessment of gene expression. Thus identifying such biomarkers remains an alluring challenge.

Herein, we performed global gene expression analysis of three phenotypically distinct wine yeast strains [8] during alcoholic fermentation under two radically different nitrogen concentrations. This comparative transcriptomic analysis revealed common and strain-specific responses to nitrogen availability. We were able to correlate physiological and transcriptome data disclosing cellular mechanisms associated with yeast performance during alcoholic fermentation. Finally, we anticipated nutrient-specific (nitrogen specific) expression pattern which will facilitate the development of nitrogen deficiency diagnosis and strategies to optimize yeast performance in industrial fermentations.

## Material and Methods

### Strains and maintenance conditions

The commercial wine yeasts strains of *S*. *cerevisiae*, QA23, Uvaferm CEG and Zimaflore VL1 were obtained from the market as active dried yeasts (Lallemand—Proenol, Portugal). Strains were collected aseptically from active dried commercial preparations, re-hydrated in sterile water with 50 g/L of glucose according to the manufacturer’s instructions (37°C, for 30 min) and inoculated into yeast peptone dextrose medium (YPD), containing 20 g/L glucose, 10 g/L peptone and 5 g/L yeast extract. Cultures were then streaked onto Wallenstein agar plates (WL) and grown at 30°C to check for purity. Pure cultures were routinely maintained at 4°C on YPD slants, and the stocks stored at -80°C with glycerol at final concentration of 40% (v/v).

### Inocula preparation and fermentation conditions

Each one of the commercial yeast strains was rehydrated according to the manufacturer’s instructions and inoculated in synthetic grape juice media at a cell count of 10^6^ CFU/mL. Model solutions based on the chemically defined grape juice medium (GJM) formulated by Henschke and Jiranek [2] were used, with some modifications. An equimolecular mixture of glucose and fructose (100:100 g/L) was used as carbon and energy source, and the yeast assimilable nitrogen (YAN) was supplied as di-ammonium phosphate (DAP). Ammonium is present in grape juice either naturally, or added by winemakers to circumvent fermentation problems due to nitrogen limitation, being considered a good or preferred nitrogen source. In this way, the use of ammonium as sole nitrogen source besides facilitating the monitoring of nitrogen consumption profile, circumvents strain-specific differences in the use of nitrogen compounds [30, 31]. The fermentation experiments were conducted in 250 ml-flasks filled to 2/3 of their volume and fitted with a side-arm port sealed and a rubber septum for anaerobic sampling; the conditions were maintained at 20°C in an orbital shaker at 120 rpm. Two extreme initial concentrations were used: 67 mg/L, a low nitrogen condition (LN) which leads to sluggish fermentation [32, 33] or 10-fold higher, 670 mg/L, a high nitrogen condition (HN), chosen in order to guarantee that nitrogen was not the limiting fermentation factor. To investigate gene expression profiles during alcoholic fermentation, cell samples for DNA macroarray analysis were obtained from each culture medium at three different points: 12, 24, and 96h after inoculation. Samples of the yeast strain CEG were also taken at 36h to be used in the analysis of the common response to nitrogen limitation, since this strain showed a delay in ammonium consumption [8] and thus would be more appropriate to be used in that analysis.

### *Saccharomyces cerevisiae* strains genotypic characterisation

In order to distinguish between the different *S*. *cerevisiae* strains from diverse geographical provenance, the strains were subjected to analysis with two methods: interdelta and microsatellite fingerprinting.

#### DNA amplification

PCR amplifications were carried out in 25 μL reaction volumes containing 5–20 ng of yeast DNA, 10 mMTris pH 9.0, 50 mMKCl, 0.1% Triton X-100, 0.2 mg ml^-1^ gelatin, 200 mM of each dNTP, 2.5 mM of MgCl_2_ and 1 μM for each oligonucleotide primer. The amplification conditions and sequences for delta 1-delta 2 and M13 are those described previously by Legras and Karst [34] and by Huey and Hall [35], respectively. Amplification reactions were performed with an Applied Biosystems thermal cycler. In the case of delta1-delta2, the following programme was used: 4 min at 95°C followed by 37 cycles of 30 s at 95°C, 30 s at 52°C and 90 s at 72°C and a finishing step of 10 min at 72°C. The programme used for the amplifications with M13 was the follow: 3 min at 94°C followed by 37 cycles of 1 min at 94°C, 2 min at 50°C, and 2 min at 72°C and a finishing step of 3 min at 72°C.

#### Electrophoresis and numerical analysis

Amplification products were separated by electrophoresis on 10 or 15 cm 2% agarose gels submitted to 100 V for 1 h in 1X TBE buffer.

BioNumerics software (version 5.0, Applied Maths) was used for the alignment of fingerprinting profiles and the determination of fragment size, as well as to calculate the similarity matrices of whole densitometric curves of DNA band patterns by using the pair-wise Pearson’s product-moment correlation coefficient. The similarity matrices were subjected to cluster analysis according to the unweighted pair group with arithmetical average (UPGMA) algorithm.

### Macroarray and expression data analysis

#### Nucleic acid extraction and labeling

Total RNA extraction and labeling by cDNA synthesis using random primers and [α-^33^P]dCTP (3,000 Ci/mmol; 10 μCi/μL) was performed as described by Alberola *et al*. [36]. The labeled cDNAs were purified by using a G50 MicroSpin column (Amersham Biosciences). Between 3x10^6^ and 5x10^6^dpm/mL of labeled cDNA was used for filter hybridization. Prehybridization, hybridization, and washing were carried out according to published protocols [36].

#### Data generation, correction, and normalization

Global gene expression analysis was performed by hybridization of nylon filters containing PCR-amplified whole or partial yeast open reading frame sequences as probes [36] and hybridization signal was measured in a Phosphorimager scanner (FLA-3000; FujiFilm). Each replicate was represented by hybridizations done with two independent membranes. Hybridization signals were quantified using ArrayVision 7.0 software (Imaging Research, Inc.), taking the artifact-removed median density (with the corresponding subtracted background) as signal (sARM). Poor or inconsistent signals were not considered for further analysis. Normalization between conditions was done using the global median method. Gene expression patterns where the minimum percentage of existing values was less than 75% were eliminated from the analysis. The remaining missing values were replaced by using the KNN-imputation method (k = 10). The array data determined in this study have been submitted to the GEO data repository (http://www.ncbi.nlm.nih.gov/geo/) under accession number GSE63187.

### Statistical analysis and functional annotation of the data

#### Analysis of variance (two-way ANOVA)

To determine the effect of the strain (genotype—G), fermentation stage (environment, E), and the interaction between genotype and environment (GEI) an analysis of variance (ANOVA) was used for each type of fermentation, LN and HN. The significance of the individual model factors was tested and it was determined if the level of gene expression was altered by fermentation stage or if it varied among strains. Significance of each factor was defined at FDR<0.05 using the Benjamini and Hochberg correction [37].

#### Hierarchical clustering of the genes classified by ANOVA with an effect of genotype (G), environment (E) and interaction of both factors (GEI) for each N level

All genes identified by two-way ANOVA which showed to be affected in their expression in LN or HN fermentations were clustered in G, E, and GEI heat maps (HCLs) with expression standardized by the populations mean for each gene.

#### Correlation of phenotypic data with gene expression

In an attempt to identify genes correlated with phenotype data in DNA macroarray experiments, an analysis of the correlation between gene expression of all samples (3 strains, 2 N levels) and phenotype parameters was carried out on Excel using XLStat and the Spearman correlation coefficient and the associated statistical test. We selected genes with correlation coefficients > 0.6 or < -0.60 and *p*-value <0.05 to control the false discovery rate (FDR) following Benjamini and Hochberg procedure [37]. Phenotypic data for the three strains used in this study stem from Barbosa *et al*. [8]. In the case of growth phenotypes, specific growth rate (μ) and nitrogen uptake rate (Nrate), we used transcriptomic samples of 24h of fermentation, which is a time point more appropriated to address these parameters, since they are mainly associated with the growth phase. To address the relationship between gene expression and fermentation phenotype, maximum fermentation rate (MFR), we carried out a correlation analysis with transcriptomic samples of 96h of the three strains. The genes correlated, either positively or negatively, with the phenotypic traits were analyzed for enrichment of functional categories using the FunSpec interpreter. Only the functional categories with *p*-value <0.001 were considered significant.

#### Comparative transcriptomic analysis of the three wine yeast strains to N-availability

In order to illustrate the diversity of the expression changes occurred in LN- relative to HN-fermentations, a color matrix of pairwise correlation for the three fermentation time points (12, 24, and 96h) and the three different yeast strains was performed. To further assess the divergence/similarity of the yeast strains responses to N-availability, we compared the up-regulated genes of each strain at each fermentation stage, either in LN or HN.

#### Common response to nitrogen limitation

Regarding the response to nitrogen limitation, the data were analyzed using Rank Product (RP) [38], as implemented in the MeV software. RP, a nonparametric method designed for experiments with a small number of replicates, was used to identify differentially expressed genes, with a *p*-value cutoff of 0.05 (clamped strain data). Based on the data presented in S1 Table, LN24 (low-nitrogen fermentation at 24h) was selected for the low-nitrogen condition for QA23 and VL1 strains and LN36 (low-nitrogen fermentation at 36h) was chosen as representative of nitrogen limitation for yeast CEG. To illustrate high nitrogen conditions, HN24 (high-nitrogen fermentation at 24 h) was selected for QA23 and VL1 strains and HN36 (high-nitrogen fermentation at 36h) for the yeast CEG. Comparisons by RP analysis were done using LN conditions of the three strains as one experimental group and the HN conditions as another experimental group, in order to identify specific genes associated with the response to nitrogen limitation. To guarantee that the genes selected were responsible only for this response, we removed the genes which reacted to changes in nitrogen concentration under HN regime, i.e. genes up-regulated in samples of 24h (QA23 and VL1 strains) and 36h (CEG) when compared to the samples of 12h of HN fermentation. Furthermore, in order to confine/strict the group of genes mostly associated with limitation of nitrogen, we only considered genes common with those up regulated under LN fermentations at 24 and 36h when compared to 12h.

To identify candidate biomarker genes for predicting nitrogen deficiency during alcoholic fermentation, analyses were restricted to the genes either up- or down-regulated, at least 3 fold. This fold change was chosen in order to turn more robust the yeast cells response.

All the gene lists/sets were analyzed for enrichment of functional categories using the FunSpec interpreter [39], available online at http://funspec.med.utoronto.ca. Only the functional categories with *p*-value <0.001 were considered significant.

## Results and Discussion

**Fig 1:**
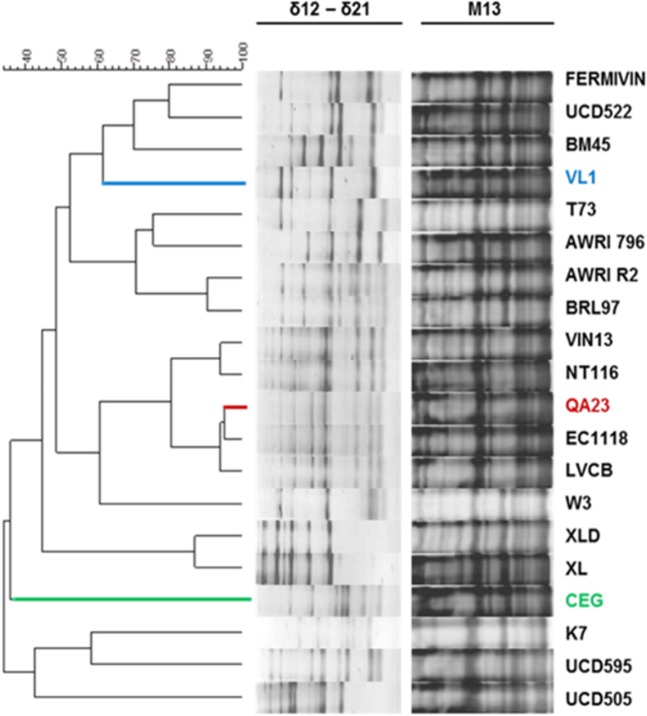
Genomic variability of VL1, CEG and QA23 yeast strains. Dendrograms obtained by composite hierarchical analysis of PCR interdelta and M13 patterns using Pearson's correlation coefficient and the UPGMA clustering method for 20 S. cerevisiae commercial selected strains. The strains grouped according to their genomic similarity. The three strains used in this study were selected on the basis of their phenotypic differences [8] and their different inter-delta and M13 PCR profiles, as shown.

In a previous study [8], we have studied the fermentative ability of eight *S*. *cerevisiae* wine strains from different geographical origins under two distinct nitrogen regimes, nitrogen limiting condition (LN) and nitrogen excessive condition (HN), in synthetic grape juice medium (GJM), which mimic wine fermentation conditions. The two nitrogen concentrations used produced large phenotypic variation for growth and fermentative activity. Based on the results obtained, we selected three phenotypically and genotypically (Fig 1) distinct wine strains for further investigation (QA23, CEG and VL1).

In the LN fermentations, the three strains presented similar fermentations profiles without significant differences for maximum fermentation rate. Nevertheless, the three strains were significantly different for maximum growth rate, production of biomass and maximum nitrogen consumption rate (VL1>QA23>CEG) (S1 Table). Contrarily to that observed in LN fermentations, in the fermentations conducted with high nitrogen levels (HN) the three strains presented dissimilar fermentations profiles, with significant differences for maximum fermentation rate (QA23>CEG>VL1). The time required by the strain QA23 to consume 200 g/L of sugar was two to threefold shorter than that required by CEG and VL1 strains, respectively. Additionally, the three strains were significantly different for maximum growth rate and biomass produced (VL1>QA23>CEG) as well as for maximum nitrogen consumption rate (QA23>CEG>VL1) [8] (S1 Table).

It is known that phenotypic diversity within species may result from phenotypic plasticity, i.e., phenotypic changes in response to environmental changes, controlled at the level of differential gene expression [40]. Thus, using the expression data of the three wine yeast strains we sought to uncover what core similarities and dissimilarities exist amongst them that could point out a set of strain specific/dependent genes that could explain the divergent growth and fermentative behaviors.

### Comparative transcriptional profiling of three wine yeast strains under two distinct nitrogen conditions

Gene expression was assessed in three wine yeast strains, CEG, VL1 and QA23, in the two fermentation conditions, LN and HN. Samples used for transcriptome profiling were taken at three time points matching the different fermentation/growth phases: the initial lag phase (12h), exponential growth phase (24h) and stationary growth-phase (96h). These conditions simulate the sequence of environments that wine yeast cells may encounter during alcoholic fermentation, including osmotic shock associated with the presence of high sugar concentration, low pH, nitrogen depletion and ethanol formation.

**Fig 2:**
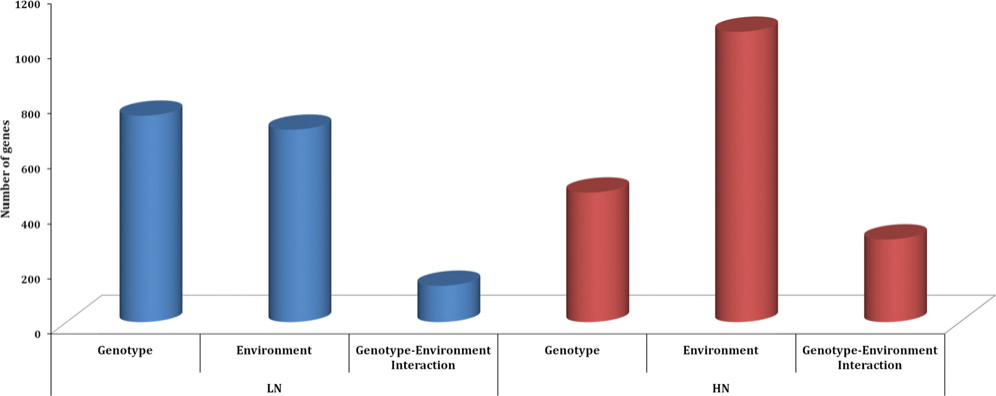
Genotype, environment, and genotype–environment interaction effects in wine yeast strains. Differentially expressed genes at each nitrogen regime studied (LN and HN), identified using two-way ANOVA. The number of genes significantly affected by genotype (strain), by environment (fermentation stage) and by the interaction between both factors is represented with bars. The selection of genes showing differential expression (with a significant effect of the different factors) was defined at FDR<0.05 using the Benjamini and Hochberg correction.

An analysis of variance (ANOVA) for each fermentation (LN and HN) was used to determine the effect of the strain (genotype—G), fermentation stage (environment—E), and the interaction between genotype and environment (GEI) on gene expression variation. The two-way ANOVA allowed us to determine how a response is affected by the two factors and whether or not they are intertwined. For the LN fermentation, of the 5764 genes analyzed, 759 genes (13%), 708 genes (12%), and 141 (2%) showed significant effects (false discovery rate (FDR) <0.05) due to genotype, environment and genotype- environment interaction, respectively. Similarly, for the HN fermentation, we identified 479 genes (8%), 1065 genes (18%), and 310 (5%) as being affected by the genotype, environment and genotype- environment interaction, respectively (Fig 2). The 141 and 310 genes which responded differently to the environments examined, depending on the strain, represent genes that are genetically variable for transcriptional plasticity [40].

The sets of genes identified as being affected by genotype and environment at both fermentation conditions (LN and HN) significantly overlap (S1 Fig). Nevertheless, the proportions of genetic differences identified within each of the fermentations were considerably different. While in the LN fermentation genotype and environment had a similar impact on gene expression variance observed, in HN fermentations the number of genes with significant effect of environment was almost two times higher than the number of genes significantly impacted by genotype. This suggests that, during LN fermentations where nitrogen is early depleted from the medium, the yeast cells adjust their growth rate to limited nutrient availability and maintain homeostasis. In this case, the transcriptional response is steadier compared to the major transcriptional response involved in the transition from active growth to stationary-growth phase coupled with higher fermentative activity. Also in the HN fermentations, the number of genes showing genotype-by-environment effect was more than twice the number of LN fermentation. The complete lists of genes significantly affected by genotype, environment and by the interaction between genotype and environment and associated GO categories are provided in S2 Table.

### Comparative transcriptomic analysis during nitrogen-limiting fermentation

**Fig 3:**
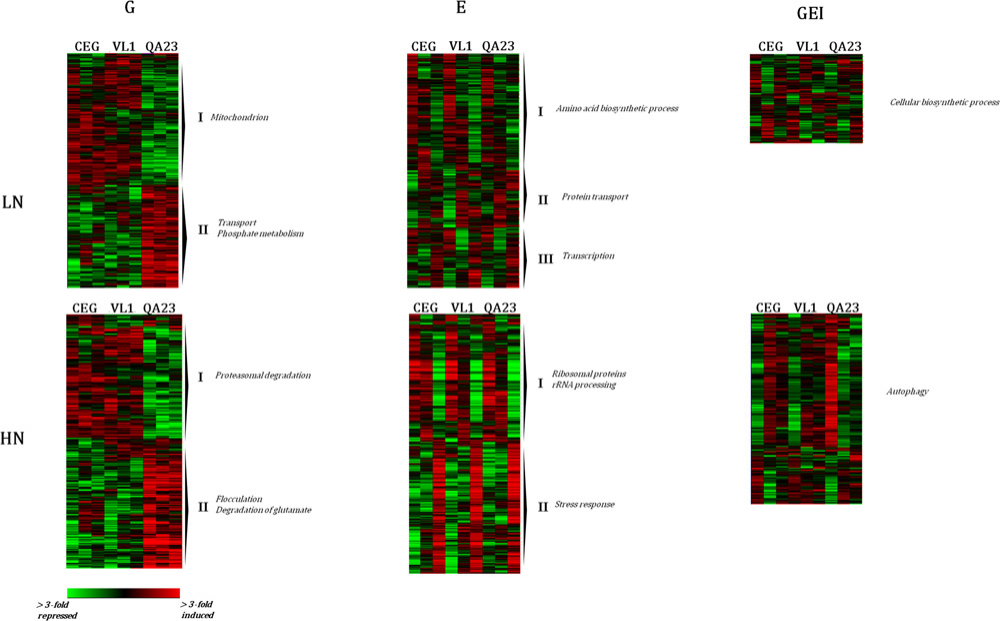
Hierarchical clustering of genes identified as exhibiting significant genotype (G), environmental (E), and gene-by-environment (GEI) effects in each of the fermentations (LN and HN). The diagrams show the log2 expression differences in the indicated strains compared to the mean expression of that gene in all strains in each fermentation. Each row represents a given gene and each column represents a different strain within which expression is ordered by fermentation stage (12, 24 and 96h). Red and green correspond to higher and lower expression, respectively. Clusters are annotated at the right with characteristic GO category functional enrichments (S2 Table).

The hierarchical clustering of the genes classified by ANOVA with significant effect of genotype (G), was able to identify strain-specific patterns of expression (Fig 3), with the majority of differences relating to QA23. Despite the significant differences on growth parameters between CEG and VL1, we found a similarity between their expression profiles.

The strain QA23 exhibited uniquely high expression of 333 genes (Cluster II) enriched for transport and phosphate metabolism throughout fermentation. Also included in this cluster we found twelve genes encoding transcription factors, including several involved in transcriptional control (*INO2*, *MGA1*, *YAP3*, *CHA4*, *HAP1*, *RTG1*, *CUP9 and ARR1)*. This variation in transcriptional regulation among wine yeast strains has been proposed to be responsible for differences and specific adaptations to different fermentative conditions [41]. QA23 was distinct from the other two strains by presenting lower expression levels of genes (cluster I) involved in mitochondrion functions including mitochondrial translation and transport. Included in these genes, and highly expressed in VL1 strain we found *AIF1*, an apoptosis-inducing factor that play a positive role in yeast longevity during winemaking [42] and *MEP1* encoding for an ammonium permease whose expression is under the nitrogen catabolite repression regulation [43], presumably reflecting the higher nitrogen consumption rate determined for VL1 in this fermentation condition, compared with the other two strains (S1 Table).

Also, the hierarchical clustering of the genes classified by ANOVA as significantly affected by environment (E), revealed fermentation stage-specific patterns of expression across strains. We found 282 genes (Cluster I) with higher expression at 12h and progressive decrease in gene expression throughout LN fermentations. This cluster is enriched for genes mainly involved in cellular amino acid biosynthetic process (P = 2.8 x 10^-6^), coinciding with nitrogen depletion from fermentation medium, and for genes involved in metabolic process, in line with the lower metabolic activity observed in these cells. Nevertheless, even though yeast were starved for nitrogen, extracellular glucose and ethanol concentrations continuously changed throughout LN fermentation, indicating that these cells continued to be metabolically active. Clusters II and III included genes with elevated expression along nitrogen depletion, mainly involved in protein transport and transcription. We also found genes related to autoproteolytic processing (*ATG1*, *ATG17*, *ATG18* and *ATG31*), respiratory metabolism (*COX9*, *PAH1*, *CYC7*), including the glucose-repressed regulator of respiratory gene expression *HAP4*. These results are in agreement those previously reported [44] that in nitrogen limiting fermentation there is an increased generation of reactive oxygen species (ROS) and up-regulation of autophagy, following nitrogen-starvation. In addition, among the genes with higher expression at 96h (cluster III) we found transcription factors such as *HAP4*, *CAT8* and *GCR1*, involved in the regulation of C-compound and carbohydrate metabolism. Our results together with those of previous studies [12, 17] indicate that there is a common response of wine yeast strains to nitrogen limitation characterized by the induction of genes involved in oxidative energy metabolism, which normally would be repressed by the high sugar concentration present, and by the repression of genes associated with fermentative metabolism. As stated, in the LN fermentations we found 141 genes dependent on GEI effect, corresponding to transcriptional responses that are highly variable among yeast strain and dependent on the fermentation stage. We found that almost 50% of these genes had no molecular function known, and the remaining cover a variety of biological processes. For instance, we found genes involved in cellular biosynthetic process (*PRS2*, *PRS12*), and involved in the TOR signaling pathway (*TCO89*), which is known to regulate yeast growth in response to nutrient availability [45, 46] and was recently identified as a fermentation essential gene [47].

### Comparative transcriptomic analysis during high nitrogen fermentation

The hierarchical clustering of 479 genes with an effect of genotype (G) identified major differences in gene expression between strains, most importantly between QA23 and the other two strains (Fig 3). Indeed, this group of genes is strikingly divided in two main clusters, comprising 233 and 246 genes that were always lower and higher expressed in QA23, respectively, in every fermentation stages.

Flocculation was the most highly over-represented category (P = 4.8 X 10^-4^) in the annotations of genes higher expressed in QA23 during HN fermentation (Cluster II). The ability to flocculate is one of the desirable characteristics that a wine strain should present [1], yet, if the onset of flocculation occurs too early, it may lead to incomplete sugar fermentation. On the other hand, *FLO* genes expression has also been correlated with changes in the general physical-chemical properties of the cell wall [48]. The activation of these adhesion-encoding genes is suggested to allow cells to adapt to stress conditions [49] and, particularly, *FLO10* is included in the Fermentation Stress Response (FSR) genes [18]. Although it is known that not all genes showing a transcriptional alterations in response to a given culture condition are required for fitness under these conditions [50, 51], it would be interesting to speculate that the higher expression of these genes in the strain QA23 could be associated with its improved fermentative behaviour. Regarding the effect of the environment (E), we were able to identify fermentation stage-specific patterns of expression, particularly two main clusters (cluster I and II), comprising 524 genes highly expressed at 12 and 24h and 540 genes highly expressed later at 96h, respectively. The former cluster was highly enriched for genes involved in ribosomal proteins (P = 6.1 X 10^-9^) and rRNA processing (P = 2.3 X 10^-7^), coinciding with the yeast growth phase and may also be one of the mechanisms by which DAP enhances yeast fermentation kinetics to ensure fast and efficient alcoholic fermentations [17, 24, 52].

The 310 genes with GEI effect in HN fermentation were enriched for autophagy (P = 9.1 X 10^-4^). Previous studies showed that genes that function in autophagy are required for optimal yeast survival during fermentation, even in a nitrogen-replete environment [53], and are important for improved fermentation fitness [47], suggesting that autophagy may be triggered by stress conditions, other than nutrient limitation, that arise during fermentation.

### Correlation of phenotypic traits with gene expression

Most experiments reported only the diverse differential expression genes without correlation with biological phenotypes, especially phenotypic data. Specific phenotypes are generally attributed to different gene expression levels. Since high-throughput measurement of gene expression levels has become possible, some studies have identified genes showing differential expression between two or more phenotypic groups with hope that these genes are responsible for the phenotypic differences [7, 24]. In this study, we intended to establish, if possible, a correlation between gene expression with the phenotypic traits concerning the yeast growth and fermentative activity, aiming to elucidate the underlying cellular physiology and to find phenotype deterministic genes. Using Spearman coefficient correlation (see Materials and Methods), we found that the expression of a large number of genes exhibited significant correlation, either positive or negative, with the kinetic parameters (S3 Table and S2 Fig).

The expression of a total of 457 genes was found to correlate with Nitrogen uptake rate (Nrate), 360 positively and 97 negatively. From these data we observed that high Nrates were associated with the overexpression of genes involved in nitrogen, sulphur and selenium metabolism; homeostasis of metal ions; vacuolar/lysosomal transport and lipid, fatty acid and isoprenoid metabolism (S3 Table and S2 Fig). Moreover, we found out that in this set of 360 genes, 11 are identified as NCR genes [54] and four as signature genes for predicting nitrogen deficiency [59].

Regarding the negatively correlated genes, they were mainly involved in ribosomal proteins (RP) (S2 Fig), demonstrating that, indeed, the ribosomal protein-encoding genes transcription is quite sensitive to the growth potential of the cell, rapidly increasing during nutrient upshifts and rapidly decreasing during nutrient downshifts or in response to a variety of stresses [55]. Based on this result, we could assume that higher the rate of nitrogen consumption, lower the amount of extracellular nitrogen available, which endorses to a decrease in the expression of RP genes, since the decrease in protein synthesis may help the cells to conserve mass and energy [20]. The response to availability of nutrients and increased growth rate is associated with up-regulation of biosynthetic capacity [55]. In this way, the genes positively correlated with the specific growth rate were mostly related with cellular import (S3 Table and S2 Fig). This set of 504 genes comprised 22 transcriptional factors, such as *DAL80*, corroborating the notion that yeast preferentially uses substrates that allow the best growth through the NCR mechanism. The genes *MEP1* and *MEP2* also showed positive correlation with growth rate attesting their role as ammonium permeases and sensors, which promote ammonium consumption to fuel yeast growth.

Genes with the strongest positive correlation with MFR were involved in stress response, C-compound and carbohydrate metabolism and metabolism of energy reserves (S2 Fig). Several of these genes were also correlated with the final biomass produced (Data not shown), indicating that higher the concentration of biomass, quicker the fermentation, in line with a previous report [56], which revealed that the rate of fermentation was a linear function of biomass. Some genes involved in stress response presented herein a strong positive correlation with the MFR (S3 Table), as previously described for maximum fermentation rate (R_max_) [56]. These results suggest that a high stress response is associated with a high fermentation capacity. Interestingly, 39 out of genes correlated with MFR were also positively correlated with Nrate, proposing that strains displaying higher Nrates have, consequently, better fermentative fitness defined by higher MFR, which was the case herein of QA23 strain under HN regime (S1 Table). Among these genes, were included *HXT11* and *HXT17*, which have role in sugars transmembrane transporter activity, and *PDR11*, involved in multiple drug resistance [57]. The correlation of genes with both MFR and Nrate highlights the role of nitrogen in the control of fermentative activity.

The negative correlation between gene expression and MFR comprised 442 genes (S3 Table), the most significant functional categories being ribosomal proteins, rRNA processing, RNA binding and ribosome biogenesis (S2 Fig). This result is not in accordance with other studies [7] who found positive correlation between these families of genes and MFR. These contrasting results are, probably, mainly due to different stages at which gene expression were analyzed. In this study the correlation between this phenotypic trait and gene expression was performed using transcriptomic samples of 96h of fermentations in both nitrogen levels, which could indicate that, at this stage, progression into stationary phase and stressful environmental lead to ESR programme, where repression of RP genes, tRNA synthesis and protein translation is a general feature [20].

Additionally, *DAL1-3*, *DAL7* and *DAL80* genes, involved in catabolism of nitrogen compounds also showed negative correlation with fermentation rate. Since *DAL* genes are proposed markers of nitrogen limitation [58], it makes sense that their expression could represent a signal of “nitrogen stress” within the cell, which leads to lower fermentation rates.

### Comparative transcriptomic analysis of the three wine yeast strains response to N-availability

**Fig 4:**
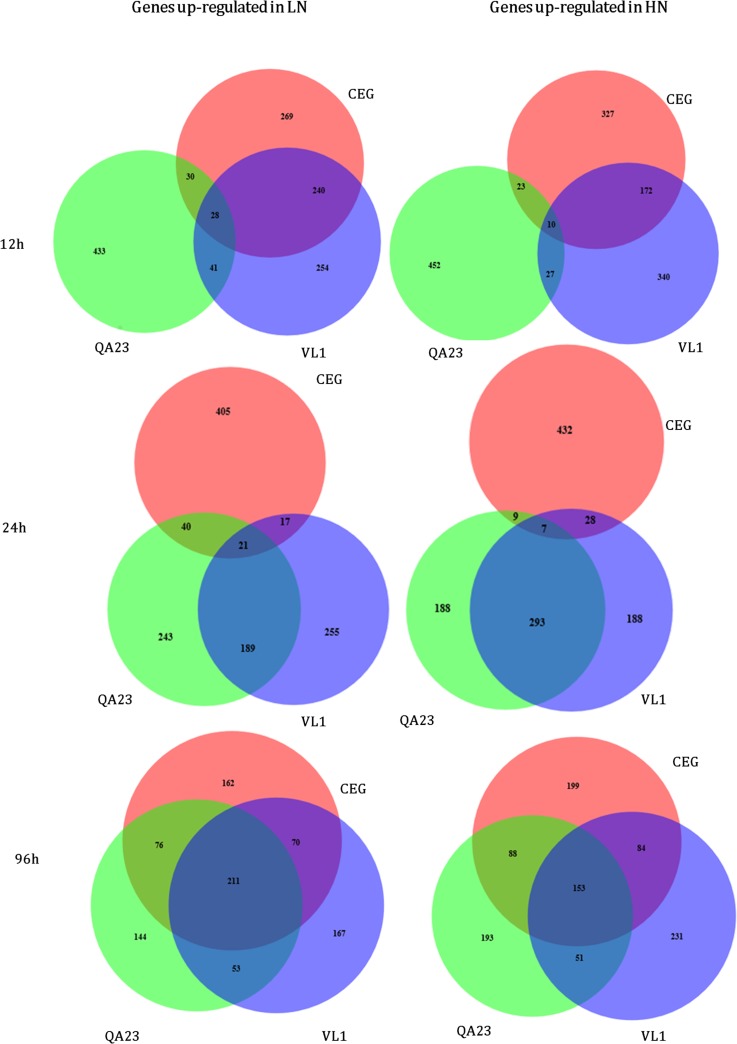
Venn diagrams for genes whose expression was significantly altered in the three wine yeast strains in LN and HN fermentations. Red indicated genes whose expression was altered in CEG, green in QA23 and violet in VL1.

Even though the strains displayed dissimilar gene expression levels across fermentation (LN and HN), we asked if there is a “conservation” of the wine yeast strains response to N-availability during fermentation. For this purpose, the significantly differentially expressed genes between conditions (LN/HN), for each strain were pair-wise compared at each fermentation stage (Fig 4 and S3 Fig). The high number of genes differentially expressed in each of the wine strains under LN and HN fermentations (Fig 4) showed that their response was distinct at each fermentation stage and within the nitrogen regimes analyzed. Maximal expression variability among the three yeast strains was reached at the initial fermentation stages in agreement with pair-wise correlation values calculated between different time points for the different strains (See S3 Fig).

Strikingly, even at 12h where the main difference between the two fermentation media was the amount of nitrogen available, that yet was non-limiting, we found a huge number of genes differentially expressed for each strain and only 28 and 10 genes commonly higher expressed in LN and HN fermentations, respectively (Fig 4). This fact suggests that the early response to nitrogen availability and the adaptation to the fermentation media differentiate winemaking strains.

At 12h, the highlighted difference between QA23 and the other two strains was confirmed by the highest number of genes differently expressed in both nitrogen conditions. The strains CEG and VL1 had relatively high similarity at the beginning of fermentation (12h), either in LN (240 genes, ~50%) or in HN (172 genes, ~35%) (See Fig 4). These two strains behaved in the same way concerning metabolism and amino acids transport when subjected to low concentrations of nitrogen, showed by the overlapping of 240 genes mainly related to amino acid/amino acid derivatives transport (P = 3.10E^-06^) (S4 Table). Interestingly, these strains showed, either individually or in common, up-regulation of genes involved in nitrogen catabolite repression (NCR), demonstrating that they are more sensitive to nitrogen than QA23, that did not present any NCR gene (S4 Table).

In the HN conditions, CEG and VL1 showed common up-regulation of genes involved in the TOR signaling cascade (S4 Table). In yeast, TOR signaling is regulated by nutrient availability [59], which was demonstrated herein, since large amounts of ammonium present in the medium prompted positively the activation of TOR cascade.

At 24h, QA23 and VL1 were the most related strains in terms of the genes differentially expressed. In LN conditions, these two strains commonly responded with 189 genes (Fig 4), which were involved in catabolism of nitrogenous compounds (P = 3.8E^-06^), metabolism of urea (P = 4.3E^-04^), oxidative stress response (P = 8.9E^-04^), metabolism of energy reserves (P = 9.9E^-04^), among others (S4 Table). The transcriptional changes represented by these functional categories seem to be a response to nitrogen limitation, since it coincided with the onset of ammonia exhaustion in both strains fermentation media. The up-regulation of genes involved in the metabolism of energy reserves demonstrates that, even in a nitrogen-starved status, the cells presented substantial metabolic activity possibly due to the carbohydrates and lipids accumulation, which was previously reported as important feature for preservation of viability and metabolic capacities during starvation [60]. Parrou and co-workers [61] also stated that complete exhaustion of the nitrogen source appears to be the primary condition for triggering the activation of both the glycogen and trehalose anabolism. Under anaerobic conditions, it is unlikely that gene products related to respiration and electron transport are functional [62]. However, genes related to these metabolic activities were up-regulated in QA23 and VL1 at 24h, under LN. Although similar observations have been reported in wine yeasts [17, 63] and sake brewing yeasts [62], the significance of the elevated expression of these genes is still unknown. Nevertheless, the response to limitation of nitrogen observed herein in regard to mitochondrial activity and oxidative metabolism is in accordance to those obtained by others, suggesting that yeast survival under alcoholic fermentation conditions could be correlated with their ability to detoxify ROS [44,64]. Among the genes commonly up-regulated at 24h in QA23 and VL1 strains under LN, there were some *ATG* genes (S4 Table), indicating that autophagy may be implicated in survival of nitrogen stress as demonstrated previously [44, 53]. Under HN conditions, QA23 and VL1 shared 293 genes (Fig 4), which were involved in growth related functions, such as ribosomal proteins, ribosome biogenesis and rRNA processing (S4 Table). The high expression of the genes involved in these biological processes at 24h under HN may also be one of the mechanisms by which DAP enhances yeast fermentation kinetics to ensure fast and efficient alcoholic fermentations [33, 44, 52], corroborating the higher growth and maximum fermentation rates observed, particularly for QA23 (S1 Table).

The yeast strain CEG, as demonstrated in a previous work [8], is significantly divergent from the other two strains in regard to growth performance, nitrogen requirements and metabolic activity, which is in line with the dissimilar transcriptional response at 24h, either in LN or in HN. Under LN regime, this strain exhibited a clear weaker nitrogen starvation response than the other two strains, essentially due to the presence of considerable amounts of ammonia in the media (S1 Table). The 405 genes up-regulated in LN (Fig 4) were mainly associated with transcriptional control, rRNA processing and ribosome biogenesis, demonstrating that yeast cells were driven the cellular response to growth related functions rather than stress response (S4 Table). Although this strain did not show the same higher growth rates of the other two strains (S1 Table), 10% of the total genes differentially expressed were related with ribosomal proteins. This lack of correlation between growth rate and ribosomal biogenesis gene expression was also observed in other study [65]. The 435 genes up-regulated by this strain in HN fermentations comprised genes involved in metabolic process (S4 Table), namely genes involved in alcohol fermentation, *ADH5*, *PDC6*, *ADH2*, *ALD4*, corroborating the higher metabolic and fermentative activity observed when larger amounts of nitrogen were available, in accordance with the higher MFR and levels of sugars present in the media (S1 Table). Furthermore, this set of 435 genes comprised much more stress genes, either ESR [20] or FSR [18] than in LN fermentation, which could be a signal that, at least for this strain, the cells underwent to a more markedly stress status under ammonium excess. Nevertheless, we did not observe cell death or stuck fermentation as reported recently by others [66], may be due to the non-limiting lipid conditions of the synthetic grape must used in our study.

At the final stage of fermentation, irrespective of nitrogen regime, the three strains converge to a similar transcriptional response demonstrated by a high degree in the overlapping of gene expression (Fig 4). Regarding LN fermentations, the common differentially expressed genes in the three strains were first and foremost ribosomal protein genes, suggesting its important role in yeast cell survival under nitrogen depletion and corroborating previous result obtained in other wine yeast [17]. Evidence from both ribosomal protein encoding genes and genes for proteins that regulate ribosome biogenesis supports an important role for ribosomal recycling in survival during starvation stress in *S*. *cerevisiae* [67]. On the contrary, the genes commonly up-regulated in the three strains under HN were mainly involved in stress response and metabolism of energy reserves (S4 Table), processes that represent a general feature of the ESR [20] and that are transcriptionally induced in cells exposed to stress conditions, such as high ethanol levels and nutrients depletion.

### Two-class Rank Product analysis reveals nitrogen-limitation specific genes

Using the expression data of the three markedly distinct wine yeast strains we sought to uncover which core similarities exist amongst them that could point to a highly conserved set of genes that responds to grape juice nitrogen availability. For this purpose, we performed a two-class Rank Product analysis grouping yeast expression data in LN fermentations, using samples collected at 24h for QA23 and VL1 and at 36h for CEG (N-limitation), then comparing their expression profiles to the corresponding nitrogen excess group (HN). The three yeast strains were still actively growing at this fermentation stage and obviously differed in their growth rates, nutritional states and gene expression patterns (S1 Table). Comparing their combined expression patterns, at the time where nitrogen was becoming exhausted in LN to that of HN fermentations were nitrogen was in excess, we uncovered classes of genes whose altered regulation could be attributed to nitrogen limitation *per se*. After the application of the criteria and using cutoffs described in Materials and Methods section, we were able to identify genes that were differentially expressed, being 190 genes up- and 554 down-regulated in nitrogen limitation conditions (S5 Table). Among the down-regulated genes in LN, the most over represented processes were involved in growth-related functions. Down regulation of these genes is part of the general yeast environmental stress response [20], being the expression of a large part of these genes highly correlated with growth rate under various medium conditions [68]. In this work, we found that expression of these ribosome-related genes did not correlate with growth-rate, as no significant differences were found among the growth rates of each strain in the two nitrogen conditions (S1 Table). The downshift on the expression of genes associated with ribosomal proteins, rRNA processing and rRNA synthesis observed in LN fermentations during exponential growth phase, in fact, can be considered as an advanced transcriptional response to the future nitrogen depletion from the medium. Under a conflicting situation between the environmental signal and yeast growth rate, the gene expression levels appears to be more linked to the environment than to yeast growth, as previously suggested [68, 69]. On the other hand, the domains of yeast metabolism related to metabolism of urea, degradation of arginine, metabolism of energy reserves and catabolism of nitrogen compounds, were heavily impacted by the differences in the nitrogen levels of the fermentation media, being up-regulated in the LN conditions. These results are in line with those obtained by Gasch and co-workers [20], where a quickly induction of genes involved in amino acid biosynthesis and allantoin utilization and initiation of ESR were observed under nitrogen limitation.

As discussed above for the common up-regulated genes at 24h in LN fermentations by QA23 and VL1, trehalose accumulation could be an indicator of nitrogen limitation conditions during fermentation. In line with this, the domain of metabolism of energy reserves comprised three genes involved in trehalose biosynthesis, *TPS1*, *TPS2* and *TSL1*. Interestingly, also up-regulated in LN, there were genes involved either in glutamate degradation I (*GAD1*, *UGA1* and *UGA2*) or in 4-aminobutyrate degradation (*UGA1* and *UGA2*) pathways, which lead to succinate formation. This result attests the higher levels of succinic acid obtained by the three strains in LN compared to HN fermentations [8].

Two *DAL* genes (*DAL*1 and *DAL*2), which are required for degradation of allantoin, a metabolite involved in purine metabolism for nitrogen recycling [69] were also induced under limitation of nitrogen, approximately 5- and 7-fold higher expressed relatively to HN condition. This result confirmed findings of a previous study in which they were proposed to be key markers of nitrogen limitation [58], corroborating that cells were growing under increased nitrogen limitation. Despite the importance of post-transcriptional and post-translational mechanisms in the regulation of arginase activity [25], under our experimental conditions, the regulation of *CAR1* expression was dependent on the nitrogen concentration, being around 23-fold up-regulated under LN (See S5 Table).

Although there are previous reports where some of the genes identified herein have been related to the consumption of nitrogen [17, 25, 29, 54], 28% of the genes (53 out of the total 190 genes) have unknown function (S5 Table), hypothesizing for the first time their participation in nitrogen metabolism and, particularly, in yeast cells response to nitrogen limitation. These potential candidates could play a role in adaptation to growth under suboptimal nitrogen conditions.

In order to select the most suitable biomarker genes for predicting nitrogen deficiency during alcoholic fermentation, analyses were restricted to the genes either up- or down-regulated, at least four fold, to turn more robust the yeast cells response. A total of 46 genes passed the stringent criteria applied, being 27 and 19 genes up- and down-regulated, respectively (Table 1). In the Table 1 is depicted the list of genes up- and down-regulated at least 4-fold in nitrogen limited conditions, their correspondent fold change, description and the overlapping with other reports. Some of the common genes identified are proposed for being molecular markers of nitrogen deficiency, with a special emphasis to *CAR1*, *ATF1*, *DUR1*,*2* and *PUT1*, which displayed the higher up-regulation and to the ORF with unknown function, *YML057C-A*, which was the most down regulated gene under limitation of nitrogen.

**Table 1:** List of genes up- and down-regulated at least 4-fold in nitrogen limited conditions, their correspondent description and the overlapping with other reports.

					Overlaping with			
	*Gene*	ORF	Fold change	Description	Mendes-Ferreira *et al*., 2007b	Godard *et al*., 2007	Wu *et al*., 2004	Gutiérrez et al, 2013
**UP-REGULATED IN N LIMITATION**	***CAR1***	YPL111W	23.5	Arginase, responsible for arginine degradation, expression responds to both induction by arginine and nitrogen catabolite repression; disruption enhances freeze tolerance		**X**		**X**
	***ATF1***	YOR377W	11.3	Alcohol acetyltransferase with potential roles in lipid and sterol metabolism; responsible for the major part of volatile acetate ester production during fermentation				
	***"DUR1*,*2"***	YBR208C	11.1	Urea amidolyase, contains both urea carboxylase and allophanate hydrolase activities, degrades urea to CO2 and NH3; expression sensitive to nitrogen catabolite repression and induced by allophanate, an intermediate in allantoin degradation		**X**		
	***PUT1***	YLR142W	10.2	Proline oxidase, nuclear-encoded mitochondrial protein involved in utilization of proline as sole nitrogen source; PUT1 transcription is induced by Put3p in the presence of proline and the absence of a preferred nitrogen source		**X**		
	***YOR292C***	YOR292C	7.7	Putative protein of unknown function; green fluorescent protein (GFP)-fusion protein localizes to the vacuole; YOR292C is not an essential gene				
	***DUR3***	YHL016C	7.7	Plasma membrane transporter for both urea and polyamines, expression is highly sensitive to nitrogen catabolite repression and induced by allophanate, the last intermediate of the allantoindegradative pathway			**X**	**X**
	***PNS1***	YOR161C	7.3	Protein of unknown function; has similarity to Torpedo californica tCTL1p, which is postulated to be a choline transporter, neither null mutation nor overexpression affects choline transport				
	***GAD1***	YMR250W	7.1	Glutamate decarboxylase, converts glutamate into gamma-aminobutyric acid (GABA) during glutamate catabolism; involved in response to oxidative stress	**X**			
	***CAR2***	YLR438W	6.8	L-ornithine transaminase (OTAse), catalyzes the second step of arginine degradation, expression is dually-regulated by allophanate induction and a specific arginine induction process; not nitrogen catabolite repression sensitive				
	***CBP4***	YGR174C	6.7	Mitochondrial protein required for assembly of cytochrome bc1 complex; interacts with the Cbp3p-Cbp6p complex and newly synthesized cytochrome b (Cobp) to promote assembly of Cobp into the cytochrome bc1 complex				
	***DAL1***	YIR027C	6.7	Allantoinase, converts allantoin to allantoate in the first step of allantoin degradation; expression sensitive to nitrogen catabolite repression		**X**		
	***YLR125W***	YLR125W	6.2	Putative protein of unknown function; mutant has decreased Ty3 transposition; YLR125W is not an essential gene				
	***YJL107C***	YJL107C	6.1	Dubious open reading frame unlikely to encode a protein, based on available experimental and comparative sequence data; partially overlaps the verified ORF BDF2/YDL070W				
	***CMK1***	YFR014C	5.6	Calmodulin-dependent protein kinase; may play a role in stress response, many Ca++/calmodulin dependent phosphorylation substrates demonstrated in vitro, amino acid sequence similar to mammalian Cam Kinase II; CMK1 has a paralog, CMK2, that arose from the whole genome duplication				
	***YSW1***	YBR148W	5.5	Protein required for normal prospore membrane formation; interacts with Gip1p, which is the meiosis-specific regulatory subunit of the Glc7p protein phosphatase; expressed specifically in spores and localizes to the prospore membrane				
	***YIR030W-A***	YIR030W-A	5.2	Dubious open reading frame; unlikely to encode a functional protein, based on available experimental and comparative sequence data				
	***YEL067C***	YEL067C	5.1	Putative protein of unknown function; the authentic, non-tagged protein is detected in highly purified mitochondria in high-throughput studies				
	***DAL2***	YIR029W	4.8	Allantoicase, converts allantoate to urea and ureidoglycolate in the second step of allantoin degradation; expression sensitive to nitrogen catabolite repression and induced by allophanate, an intermediate in allantoin degradation		**X**		
	***ADY3***	YDL239C	4.6	Protein required for spore wall formation, thought to mediate assembly of a Don1p-containing structure at the leading edge of the prospore membrane via interaction with spindle pole body components; potentially phosphorylated by Cdc28p				
	***SDP1***	YIL113W	4.6	Stress-inducible dual-specificity MAP kinase phosphatase, negatively regulates Slt2p MAP kinase by direct dephosphorylation, diffuse localization under normal conditions shifts to punctate localization after heat shock	**X**			
	***AIM17***	YHL021C	4.5	Putative protein of unknown function; the authentic, non-tagged protein is detected in highly purified mitochondria in high-throughput studies; null mutant displays reduced frequency of mitochondrial genome loss				
	***AGX1***	YFL030W	4.4	Alanine:glyoxylate aminotransferase (AGT); catalyzes the synthesis of glycine from glyoxylate, which is one of three pathways for glycine biosynthesis in yeast; has similarity to mammalian and plant alanine:glyoxylate aminotransferases				
	***HSP12***	YFL014W	4.2	Plasma membrane protein involved in maintaining membrane organization in stress conditions; induced by heat shock, oxidative stress, osmostress, stationary phase, glucose depletion, oleate and alcohol; regulated by HOG and Ras-Pka pathways				
	***PUT4***	YOR348C	4.2	Proline permease, required for high-affinity transport of proline; also transports the toxic proline analog azetidine-2-carboxylate (AzC); PUT4 transcription is repressed in ammonia-grown cells		**X**		
	***OLI1***	Q0130	4.0	F0-ATP synthase subunit c (ATPase-associated proteolipid), encoded on the mitochondrial genome; mutation confers oligomycin resistance; expression is specifically dependent on the nuclear genes AEP1 and AEP2				
	***FMP33***	YJL161W	4.0	Putative protein of unknown function; the authentic, non-tagged protein is detected in highly purified mitochondria in high-throughput studies				
	***DCG1***	YIR030C	4.0	Protein of unknown function, expression is sensitive to nitrogen catabolite repression and regulated by Dal80p; contains transmembrane domain		**X**		
**DOWN-REGULATED IN N LIMITATION**	***YML057C-A***	YML057C-A	6.4	Dubious open reading frame; unlikely to encode a functional protein, based on available experimental and comparative sequence data; overlaps the verified gene CMP2/YML057W				
	***RRP3***	YHR065C	6.0	Protein involved in rRNA processing; required for maturation of the 35S primary transcript of pre-rRNA and for cleavage leading to mature 18S rRNA; homologous to eIF-4a, which is a DEAD box RNA-dependent ATPase with helicase activity				
	***MET14***	YKL001C	5.8	Adenylylsulfate kinase; required for sulfate assimilation and involved in methionine metabolism				
	***SPS100***	YHR139C	5.7	Protein required for spore wall maturation				
	***RKI1***	YOR095C	5.4	Ribose-5-phosphate ketol-isomerase; catalyzes the interconversion of ribose 5-phosphate and ribulose 5-phosphate in the pentose phosphate pathway; participates in pyridoxine biosynthesis				
	***PPR1***	YLR014C	5.4	Zinc finger transcription factor; contains a Zn(2)-Cys(6) binuclear cluster domain, positively regulates transcription of URA1, URA3, URA4, and URA10, which are involved in de novo pyrimidine biosynthesis, in response to pyrimidine starvation; activity may be modulated by interaction with Tup1p				
	***VBA2***	YBR293W	4.9	Permease of basic amino acids in the vacuolar membrane				
	***PRP42***	YDR235W	4.8	U1 snRNP protein involved in splicing; required for U1 snRNP biogenesis; contains multiple tetriatricopeptide repeats				
	***RPA135***	YPR010C	4.7	RNA polymerase I second largest subunit A135				
	***UTP13***	YLR222C	4.3	Nucleolar protein; component of the small subunit (SSU) processome containing the U3 snoRNA that is involved in processing of pre-18S rRNA				
	***MET5***	YJR137C	4.3	Sulfite reductase beta subunit; involved in amino acid biosynthesis, transcription repressed by methionine				
	***SAS5***	YOR213C	4.3	Subunit of the SAS complex (Sas2p, Sas4p, Sas5p); acetylates free histones and nucleosomes and regulates transcriptional silencing; stimulates Sas2p HAT activity				
	***YMR193C-A***	YMR193C-A	4.2	Dubious open reading frame; unlikely to encode a functional protein, based on available experimental and comparative sequence data				
	***EDC3***	YEL015W	4.2	Non-essential conserved protein with a role in mRNA decapping; specifically affects the function of the decapping enzyme Dcp1p; mediates decay of the RPS28B mRNA via binding to both Rps28Bp (or Rps28Ap) and the RPS28B mRNA; mediates decay of the YRA1 mRNA by a different, translation-independent mechanism; localizes to cytoplasmic mRNA processing bodies; forms cytoplasmic foci upon DNA replication stress				
	***CBP1***	YJL209W	4.1	Mitochondrial protein, regulator of COB mRNA stability and translation; interacts with the 5'-untranslated region of the COB mRNA; found in a complex at the inner membrane along with Pet309p; localizes to mitochondrial foci upon DNA replication stress				
	***PPT1***	YGR123C	4.1	Protein serine/threonine phosphatase; regulates Hsp90 chaperone by affecting its ATPase and cochaperone binding activities; has similarity to human phosphatase PP5; present in both the nucleus and cytoplasm; expressed during logarithmic growth				
	***LIA1***	YJR070C	4.0	Deoxyhypusine hydroxylase; HEAT-repeat containing metalloenzyme that catalyzes hypusine formation; binds to and is required for the modification of Hyp2p (eIF5A); complements S. pombe mmd1 mutants defective in mitochondrial positioning; protein abundance increases in response to DNA replication stress				
	***RPL22a***	YLR061W	4.0	Ribosomal 60S subunit protein L22A; required for the oxidative stress response in yeast; homologous to mammalian ribosomal protein L22, no bacterial homolog; RPL22A has a paralog, RPL22B, that arose from the whole genome duplication				
	***HXT13***	YEL069C	4.0	Hexose transporter; induced in the presence of non-fermentable carbon sources, induced by low levels of glucose, repressed by high levels of glucose; HXT13 has a paralog, HXT17, that arose from a segmental duplication				

Four of the genes up-regulated in LN have been classified as signature genes for predicting nitrogen deficiency [29], 9 are NCR sensitive genes [54], 5 genes are included in the Top50 ORFs induced by ammonium starvation [70] and two were identified as biomarkers for detecting nitrogen deficiency [27]. *CAR1* was the gene with the highest fold-change, being 23 fold up-regulated under nitrogen limitation (Table 1). This fact led us to, in line with previous studies [25, 27] reinforce the proposal to make use of this gene as a good marker to detect nitrogen limitation in grape must fermentations. Additionally, we found another up-regulated gene, *ADY3*, which was never associated before to nitrogen limitation conditions, representing one of the novelties of this study. In attempt to define whose of these potential biomarker genes are in fact real signature genes for predicting nitrogen limitation, further studies will be performed with different wine yeasts and nitrogen conditions.

## Conclusions

Here we provide the first genome-wide study examining genetic variation for transcriptional plasticity to environmental perturbation associated with nitrogen availability, allowing us to highlight the transcriptional differences between industrial wine strains and also to associate some gene regulatory systems that affect nitrogen assimilation and growth rates, as well as with fermentative fitness.

The results show that gene expression is highly variable among wine yeast strains. Such variability is observed throughout the range of metabolic changes faced by yeast during alcoholic fermentation. The variability in expression levels of many genes impacted key aspects of yeast metabolism and can be seen as a possible basis of phenotypic diversity dissimilarities in gene expression during fermentation affected co-regulated genes and distinguished yeast strains, which suggests that gene expression changes are not only a result of the vigour of the stress, but are also determined by the genetic background of the strain. Therefore, a particular study of cellular responses, transcriptomes and phenotypic traits is essential to be done with each new studied wine yeast strain. This study shows that the domains of yeast metabolism related to nitrogen and sulphur (including amino acid metabolism and catabolism of nitrogen compounds) are heavily impacted by the differences in composition of fermentation medium, but also highlights the impact of yeast strain identity, since VL1 presented those domains overexpressed relative to the other two strains, corroborating its high nitrogen demander character.

Altogether, these results suggest that the adaptation of the yeast strains to both nitrogen environments takes place in a different manner, justifying the specific fermentative and metabolic behavior of them [8]. Thus, at face value, the observed divergence may indicate that yeasts deal with stresses differently, suggesting that they may in fact use a similar set of genes to cope with stresses, but the expression patterns of these genes varied through fermentation course.

The fact that the QA23 yeast strain induced less-pronounced changes in its transcriptome than the two other strains, either in the number of genes and the magnitude of changes, when comparing the fermentations with contrasting nitrogen regimes, might indicate a higher degree of adaptation of this strain for alcoholic fermentation, although this remains speculative.

A key feature of our approach is to compare and contrast strains with unique phenotypes, to quickly implicate the genetic basis of the phenotypic difference in the response to nitrogen availability. This study resulted on the improvement in identifying relevant genes resulted not only from the cross-strain comparison, but also from assaying the phenotype most dependent on gene expression changes caused by N-availability. To our knowledge it was the first time that a correlation between gene expression and phenotypic traits concerning growth and nitrogen uptake rates was carried out. The correlations identified herein underlie the role of nitrogen in yeast growth rate and fermentative fitness, revealing that a high stress response is associated with a high fermentation capacity.

## Supporting Information

S1 Fig Overlap of genes dependent on, at both N regimes, genotype (G), environment (E) and genotype-environment interaction (GEI) effects. (TIF)

S2 Fig Correlation between gene expression and phenotypic traits. FunSpec functional category enrichment of genes positively (A, C and E) and negatively correlated (B, D and F) with nitrogen assimilation rate (Nrate), specific growth rate (μ) and maximum fermentation rate (MFR), respectively. The values correspond to the percentage of genes from the input cluster in given category and the p-values are indicated for each one.
(TIF)

S3 Fig Divergence of the yeast strains response to nitrogen availability. Correlation matrix for the log_2_-transformed expression responses (LN/HN) of genes in each comparison. Color matrix of pairwise correlation for the three different yeast strains (QA23, VL1, and CEG) at the three fermentation time points (12, 24, and 96h) based on the correlation coefficient.
(TIF)

S1 Table Fermentation parameters evaluated during experiments carried out in synthetic grape juice medium with different initial nitrogen regimes, low nitrogen (LN) and high nitrogen (HN). (DOCX)

S2 Table The complete lists of genes showing genotype-, environment—and interaction between genotype and environment effect and associated GO categories. (XLSX)

S3 Table Lists of genes showing significant correlation, either positive or negative, with the phenotypic traits. (XLSX)

S4 Table The complete lists of genes up-regulated in LN and HN fermentations for the three yeast strains at each time point, and associated GO categories. (XLSX)

S5 Table Lists of genes up- and down-regulated in nitrogen limitation and corresponding fold-changes. (XLSX)
