# Supplementary figures and images for: Comparative Transcriptomic Analysis Reveals Similarities and Dissimilarities in Saccharomyces cerevisiae Wine Strains Response to Nitrogen Availability
Source: PLoS One. 2015 Apr 17;10(4):e0122709. doi: 10.1371/journal.pone.0122709 (PMC4401569; doi:10.1371/journal.pone.0122709)

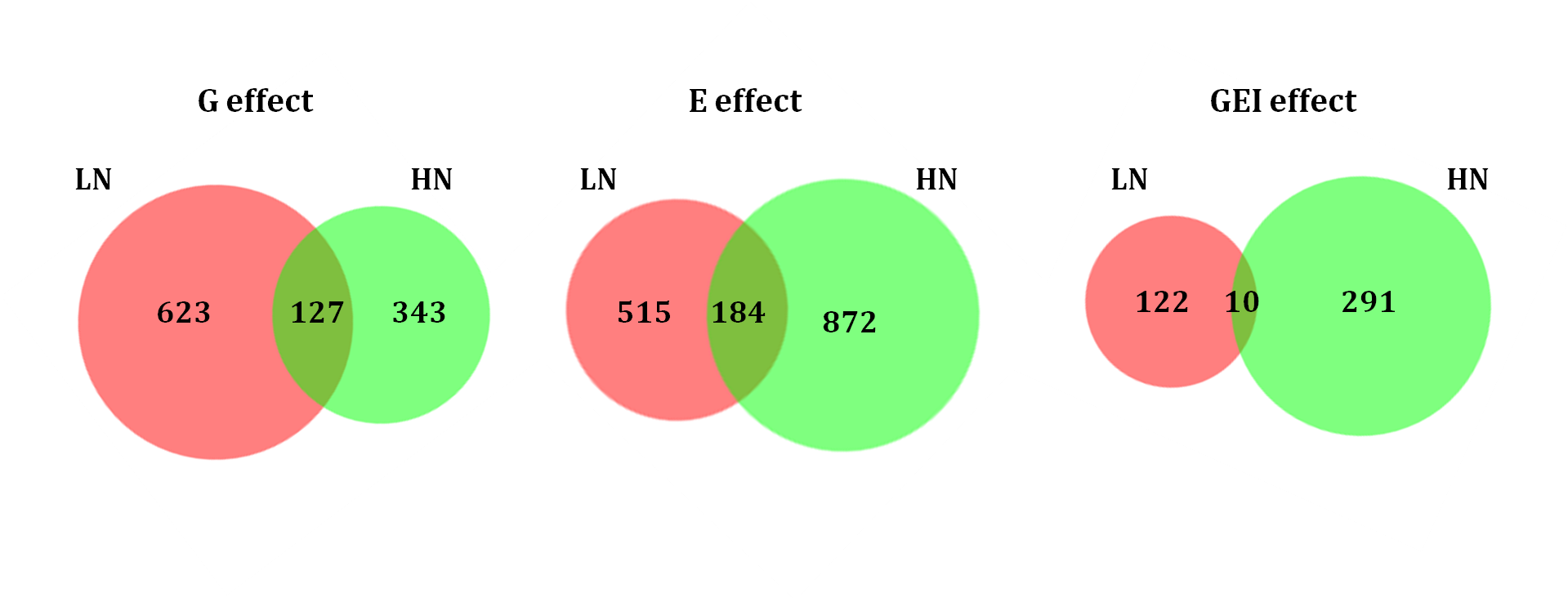

Supplement: S1 Fig — (TIF) [file pone.0122709.s001.tif]

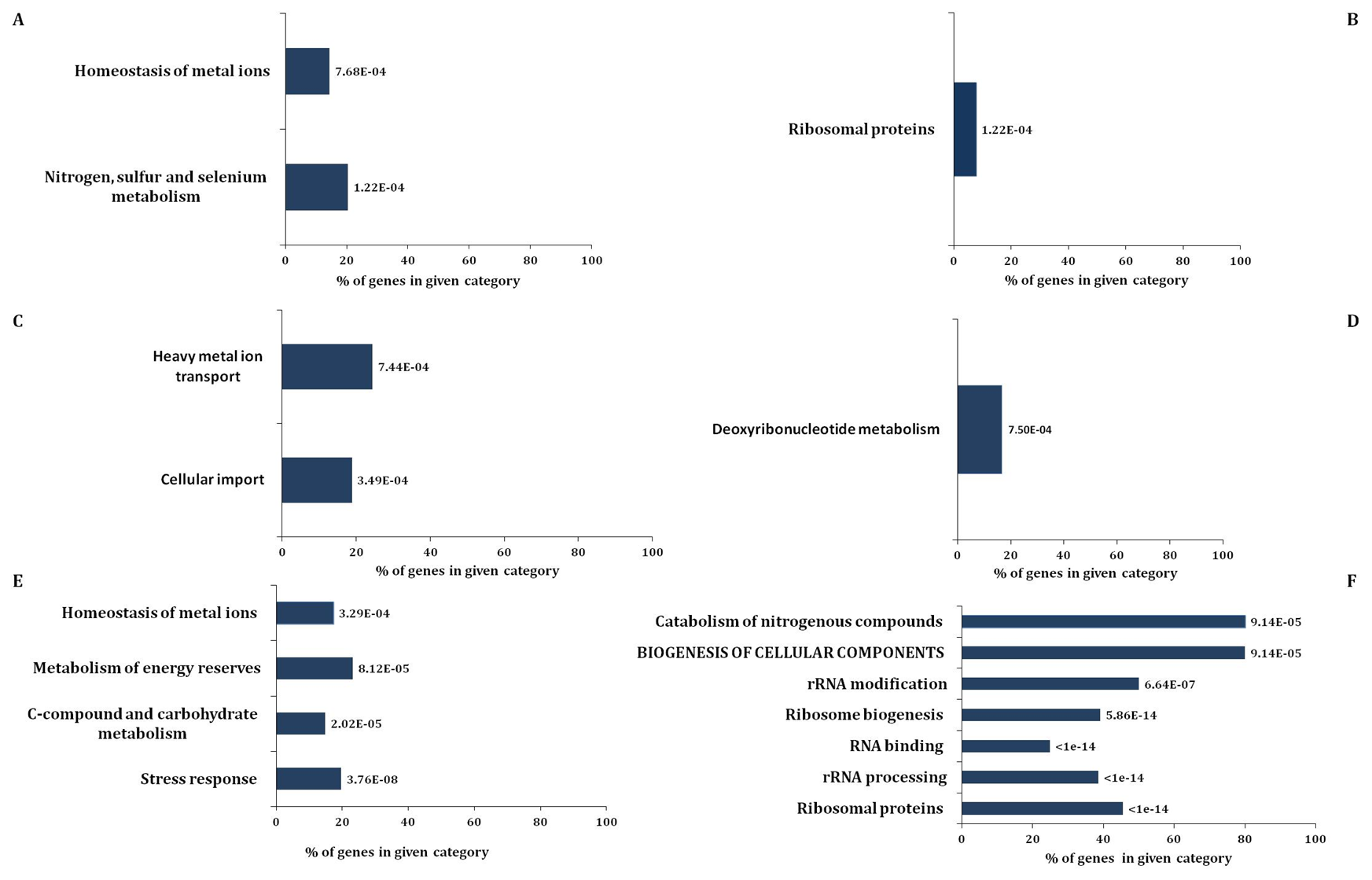

Supplement: S2 Fig — FunSpec functional category enrichment of genes positively (A, C and E) and negatively correlated (B, D and F) with nitrogen assimilation rate (Nrate), specific growth rate (μ) and maximum fermentation rate (MFR), respectively. The values correspond to the percentage of genes from the input cluster in given category and the p-values are indicated for each one. (TIF) [file pone.0122709.s002.tif]

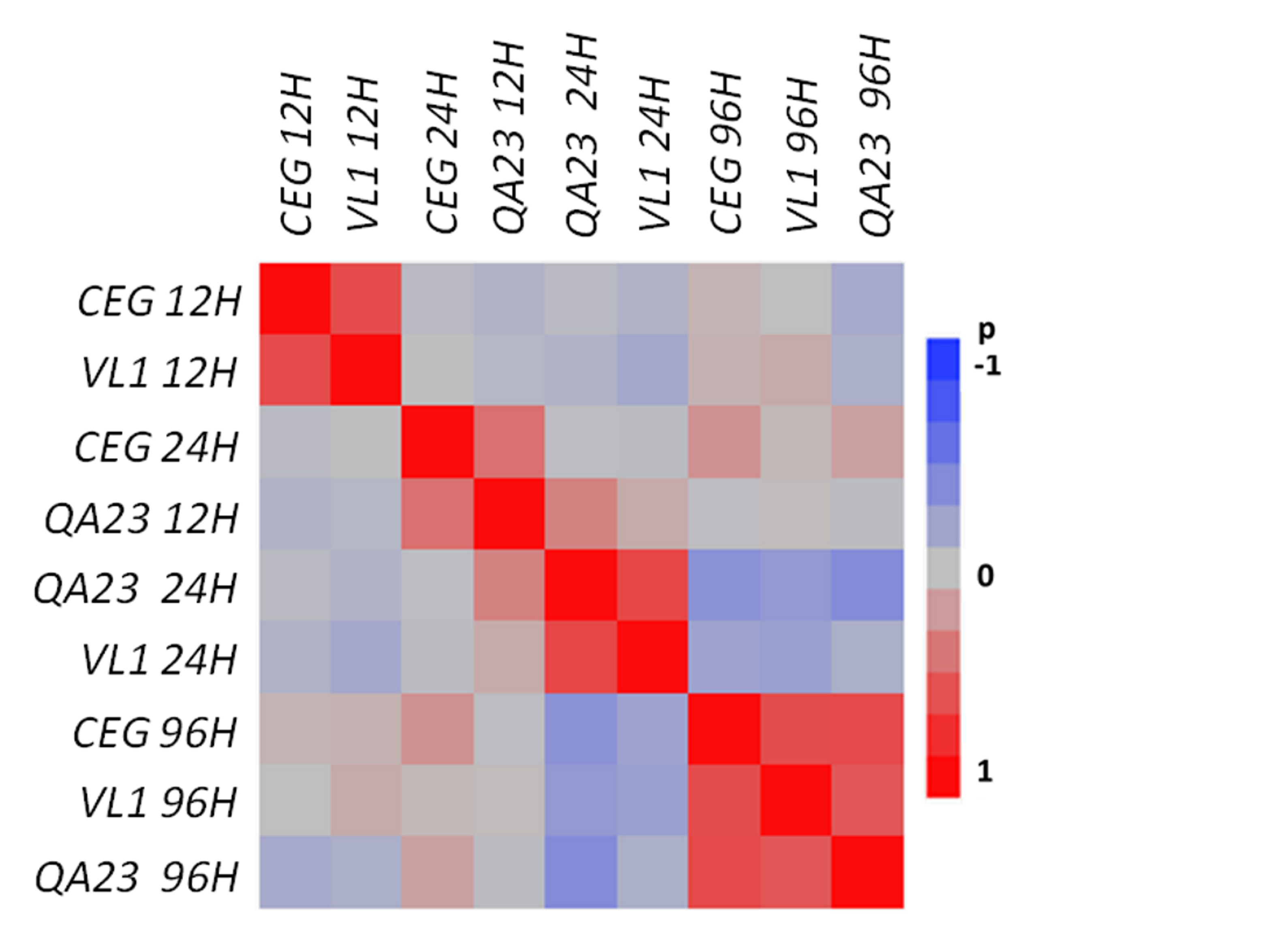

Supplement: S3 Fig — Correlation matrix for the log2-transformed expression responses (LN/HN) of genes in each comparison. Color matrix of pairwise correlation for the three different yeast strains (QA23, VL1, and CEG) at the three fermentation time points (12, 24, and 96h) based on the correlation coefficient. (TIF) [file pone.0122709.s003.tif]
