# Supplementary material for: Comparative Transcriptomic Analysis Reveals Similarities and Dissimilarities in Saccharomyces cerevisiae Wine Strains Response to Nitrogen Availability
Source: PLoS One. 2015 Apr 17;10(4):e0122709. doi: 10.1371/journal.pone.0122709 (PMC4401569; doi:10.1371/journal.pone.0122709)
Supplement: S1 Table — (DOCX) [file pone.0122709.s004.docx]

**Table S1 –** Fermentation parameters evaluated during experiments carried out in synthetic grape juice medium with different initial nitrogen regimes, low nitrogen (LN) and high nitrogen (HN).

| **Strain** | **Experiment** | **time (h)** | **Glucose (g/L)** | **Fructose (g/L)** | **Nitrogen (mg/L)** | **Ethanol (% v/v)** | **Specific growth rate μ (h^−1^)** | **Maximum fermentation rate MFR (g h^−1^)** | **Maximum nitrogen consumption rate Nrate (mg h^−1^)** |
| --- | --- | --- | --- | --- | --- | --- | --- | --- | --- |
|  |  |  |  |  |  |  |  |  |  |
| QA23 | LN | 12 | 94.79 ± 0.94 | 94.93 ± 0.30 | 45.20 ± 0.20 | 0.00 ± 0.00 | 0.170 ± 0.00 | 0.04 ± 0.01 | 3.7 ± 0.07 |
|  |  | 24 | 91.17± 2.51 | 94.44 ± 1.99 | 1.20 ± 0.70 | 0.64 ± 0.02 |  |  |  |
|  |  | 96 | 66.24 ± 6.47 | 81.03 ± 8.48 | 0.00 ± 0.00 | 3.08 ± 0.23 |  |  |  |
|  | HN | 12 | 95.6 ± 2.94 | 96.6 ± 2.94 | 596.80 ± 94.90 | 0.00 ± 0.00 | 0.173 ± 0.01 | 0.17 ± 0.01 | 5.0 ± 0.38 |
|  |  | 24 | 92.25 ± 2.38 | 95.36± 2.17 | 584.70 ± 23.00 | 0.46 ± 0.002 |  |  |  |
|  |  | 96 | 3.55 ± 1.59 | 12.37 ± 1.12 | 288.30 ± 12.60 | 10.77 ± 0.15 |  |  |  |
| VL1 | LN | 12 | 98.4 ± 1.51 | 98.41 ± 1.91 | 58.20 ± 3.90 | 0.00 ± 0.00 | 0.210 ± 0.01 | 0.04 ± 0.01 | 4.8 ± 0.31 |
|  |  | 24 | 95.70 ± 7.38 | 98.27 ± 4.31 | 1.02 ± 0.10 | 0.28 ± 0.38 |  |  |  |
|  |  | 96 | 78.12 ± 6.80 | 83.53 ± 9.43 | 0.00 ± 0.00 | 1.42 ± 0.95 |  |  |  |
|  | HN | 12 | 100.02 ± 1.82 | 99.69 ± 1.49 | 642.30 ± 32.40 | 0.00 ± 0.00 | 0.220 ± 0.00 | 0.08 ± 0.01 | 2.1 ± 0.12 |
|  |  | 24 | 92.41 ± 0.57 | 95.17 ± 0.38 | 588.20 ± 11.50 | 0.73 ± 0.24 |  |  |  |
|  |  | 96 | 26.21 ± 3.35 | 55.67 ± 1.51 | 486.30 ± 73.30 | 6.91 ± 0.28 |  |  |  |
| CEG | LN | 12 | 99.68 ± 1.24 | 99.19 ± 1.13 | 61.50 ± 1.60 | 0.00 ± 0.00 | 0.153 ± 0.00 | 0.04 ± 0.00 | 2.1 ± 0.11 |
|  |  | 24 | 97.27 ± 0.82 | 97.87 ± 0.69 | 47.40 ± 1.80 | 0.28 ± 0.09 |  |  |  |
|  |  | 36 | 91.77 ± 2.65 | 94.23 ± 1.56 | 20.10 ± 3.10 | 0.64 ± 0.12 |  |  |  |
|  |  | 96 | 71.13 ± 3.26 | 83.38 ± 3.22 | 0.00 ± 0.00 | 2.66 ± 0.38 |  |  |  |
|  | HN | 12 | 98.09 ± 2.48 | 96.42 ± 1.77 | 663.94 ± 6.78 | 0.00 ± 0.00 | 0.146 ± 0.00 | 0.13 ± 0.01 | 2.9 ± 0.12 |
|  |  | 24 | 94.02 ± 2.04 | 93.64 ± 2.04 | 650.10 ± 31.42 | 0.49 ± 0.03 |  |  |  |
|  |  | 36 | 87.97 ± 5.60 | 90.51 ± 3.70 | 636.50 ± 19.17 | 1.26 ± 0.12 |  |  |  |
|  |  | 96 | 26.73 ± 3.16 | 48.80 ± 3.86 | 391.60 ± 12.00 | 7.28 ± 0.41 |  |  |  |

Phenotypic data, μ, Nrate and MFR, stem from Barbosa *et al.*, 2014.
